# The Retentive Strength of Cemented Zirconium Oxide Crowns after Dentin Pretreatment with Desensitizing Paste Containing 8% Arginine and Calcium Carbonate

**DOI:** 10.3390/ijms17040426

**Published:** 2016-03-25

**Authors:** Raphael Pilo, Noga Harel, Joseph Nissan, Shifra Levartovsky

**Affiliations:** Department of Oral Rehabilitation, The Maurice and Gabriela Goldschleger School of Dental Medicine, Tel Aviv University, Tel Aviv 6997801, Israel; noga.hrl@gmail.com (N.H.); nissandr@gmail.com (J.N.); shifralevartov@gmail.com (S.L.)

**Keywords:** desensitizing paste, dentin, retention, cements, Y-TZP

## Abstract

The effect of dentin pretreatment with Desensitizing Paste containing 8% arginine and calcium carbonate on the retention of zirconium oxide (Y-TZP) crowns was tested. Forty molar teeth were mounted and prepared using a standardized protocol. Y-TZP crowns were produced using computer-aided design and computer-aided manufacturing (CAD-CAM) technology. The 40 prepared teeth were either pretreated with Desensitizing Paste or not pretreated. After two weeks, each group was subdivided into two groups, cemented with either Resin Modified Glass Ionomer Cement (RMGIC) or Self Adhesive Resin Cement (SARC)). Prior to cementation, the surface areas of the prepared teeth were measured. After aging, the cemented crown-tooth assemblies were tested for retentive strength using a universal testing machine. The debonded surfaces of the teeth and crowns were examined microscopically at 10× magnification. Pretreating the dentin surfaces with Desensitizing Paste prior to cementation did not affect the retention of the Y-TZP crowns. The retentive values for RMGIC (3.04 ± 0.77 MPa) were significantly higher than those for SARC (2.28 ± 0.58 MPa). The predominant failure modes for the RMGIC and SARC were adhesive cement-dentin and adhesive cement-crown, respectively. An 8.0% arginine and calcium carbonate in-office desensitizing paste can be safely used to reduce post-cementation sensitivity without reducing the retentive strength of Y-TZP crowns.

## 1. Introduction

Post-operative sensitivity often occurs after the cementation of fixed partial dentures (FPDs) [1]. Factors related to post-cementation sensitivity include aggressive tooth preparation, inadequate provisional restorations, and removal of the smear layer by acid etching, and type of cement, and there is an inverse relationship between sensitivity and patient age [2]. Several substances and methods have been suggested for reducing hypersensitivity, such as immediate dentin sealing [3]. The application of desensitizers to the prepared abutment teeth may be very effective in relieving post-cementation sensitivity for FPDs and is beneficial in terms of patient comfort [4]. Several agents have been used prior to cementation to decrease post-cementation sensitivity. These agents include dentin primers, such as an aqueous solution of 35% hydroxyethyl methacrylate (HEMA), an aqueous mixture of 35% HEMA and 5% glutaraldehyde, potassium nitrate, 5% *N*-methacryloyl-5-aminosalicylic acid diluted in ethanol, and an aqueous solution of 35% glyceryl methacrylate [5,6]. Toothpastes containing 8% arginine in combination with calcium carbonate or 2% potassium ion and strontium have also been advocated [7]. However, the literature is inconsistent regarding the effects of these agents on the retentive strength of FPDs, and there are reports of significant interactions between specific agents and cements [8,9,10]. For example, zinc phosphate cement (ZPC) was negatively influenced by All-Bond desensitizer, One-Step sealer and Gluma desensitizer, whereas Imperva bonding agent had no effect on ZPC [8,10,11]. On the other hand, One-Step sealer increased the retention of glass ionomer cement by 42% [11], All-Bond 2 desensitizer had no effect, and Gluma desensitizer reduced the retention by 16% [10]. Because retention values likely depend on the combined effects of the desensitizing agent, cement and crown type, evidence-based studies are needed to determine the benefits of specific combinations.

Over the last few years, there has been a tremendous shift toward high-strength, all-ceramic crowns, particularly those fabricated from zirconium oxide ceramics (Y-TZP), because of their excellent esthetic, mechanical and biocompatibility properties. The strategies for cementing Y-TZP to human dentin include adhesive, self-adhesive (SARC) and conventional cementation. The retention of Y-TZP ceramic crowns was tested after cementation with composite resin cement with an adhesive agent, resin-modified glass ionomer cement (RMGIC) and SARC [12]. All three types of cement were capable of retaining Y-TZP crowns successfully, and there were no significant differences among them. Alves *et al.* [13] also reported that the cement type did not affect the retention values, while surface conditioning of Y-TZP ceramics did have an effect.

Recently, a new in-office desensitizing paste containing 8% arginine and calcium carbonate was shown to provide immediate and lasting relief of dentin hypersensitivity [14,15,16,17,18]. The influence of this pretreatment was studied on enamel prior to adhesive application for composite restorations and was found to have an insignificant effect on the shear bond strength of composite to enamel [19]. Extended application times of 18 and 30 s showed no adverse effect on the bonding performance to dentin when using self-etching adhesives containing functional monomers that include 4-MET monomer (4-metacryloxyethyl trimellitic acid), such as G-Bond (GC) [20]. In addition, there was no significant difference in the bond strength of composite to dentin pretreated with an 8.0% arginine and calcium carbonate desensitizing toothpaste or pumice [21]. The application of these desensitizing toothpastes had a positive but not significant influence on the bond strength of resin cement to dentin, as evaluated with the micro-shear test [7]. However, the literature lacks information on the influence of dentin pretreatment with 8.0% arginine and calcium carbonate desensitizing paste on the retentive strength of Y-TZP crowns cemented to human teeth.

The aim of this *in vitro* study was to evaluate the effect of dentin pretreatment with Colgate Sensitive Pro-Relief Desensitizing Paste, which contains 8% arginine and calcium carbonate, on the retentive strength of Lava Y-TZP crown copings cemented with either conventional or self-adhesive cement. The null hypotheses were as follows: (1) the retentive strength of Lava Y-TZP crown copings cemented to human extracted teeth would not be affected by Colgate Sensitive Pro-Relief Desensitizing Paste, and (2) the retentive strengths of the two cements would be similar.

## 2. Results

The means (SD) of the retentive strengths in all cementation groups are presented in Table 1. Pretreating the dentin surfaces with Colgate Sensitive Pro-Relief Desensitizing Paste prior to cementation with either RMGIC or SARC did not affect the retentive strength of the Lava Y-TZP crown copings (*p* = 0.573). The retentive values for RelyX Luting 2 (RMGIC) were significantly higher than those for RelyX U-200 (SARC) (*p* = 0.001). The interaction between the cement and the dentin treatment was not significant (*p* = 0.673).

**Figure 1:**
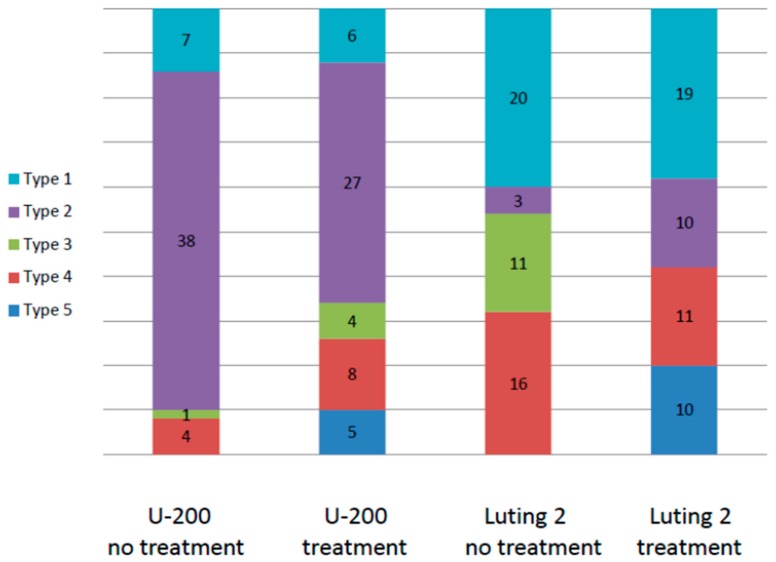
Distribution of failure modes (number of surfaces) for each cementation group: Type 1—Adhesive cement-dentin; Type 2—Adhesive cement-crown; Type 3—Cohesive cement; Type 4—Mixed mode; Type 5—Cohesive dentin.

Examining the failure mode with light microscopy after debonding revealed that the predominant failure mode for SARC was adhesive cement-crown. In 77% of the surfaces in this group, all (65%) or part (12%) of the dentin was covered with cement, and only a few remnants were detected on the crown (Figure 1). This mode of failure was more pronounced for the non-treated group. For RMGIC, the failure mode was predominantly adhesive cement-dentin. In 66% of the surfaces, all (39%) or part (27%) of the crown was covered with cement, and much less was detected on the dentin. The failure mode remained the same regardless of whether the dentin was pretreated with Colgate Sensitive Pro-Relief Desensitizing Paste.

**Figure 2:**
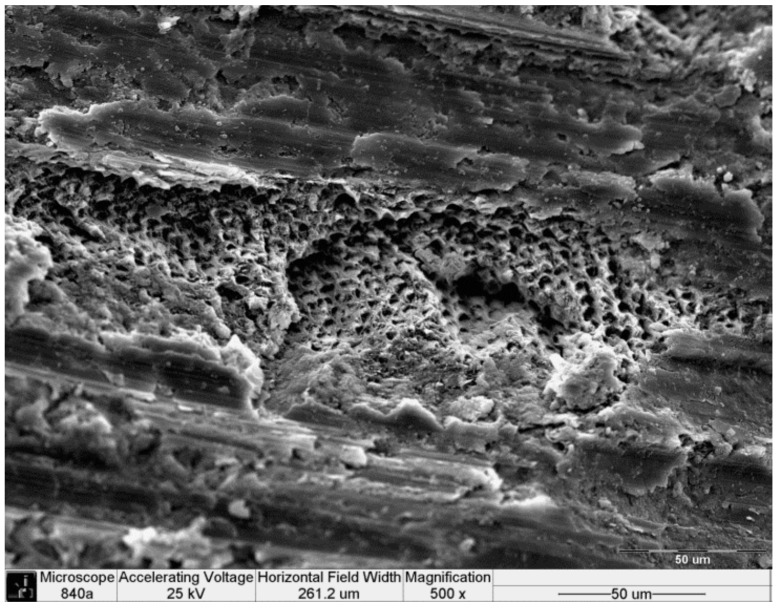
Scanning electron microscopy (SEM) micrograph (500×) of the dentin surface after preparation with a diamond bur. The longitudinal striations of the bur, open dentinal tubules and remnants of the smear layer are evident.

**Figure 3:**
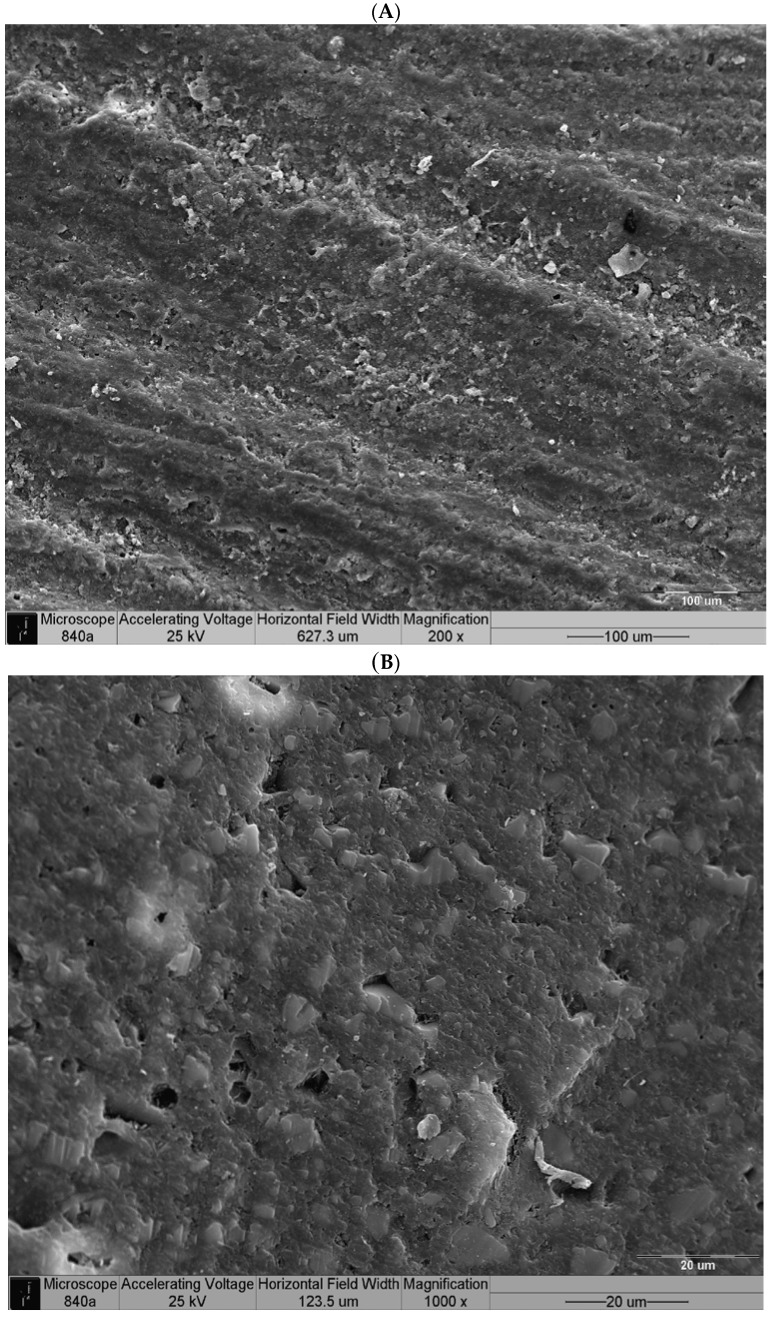
Scanning electron microscopy (SEM) micrograph of the dentin surface after pretreatment with desensitizing paste containing 8% arginine and calcium carbonate and incubation for 2 weeks: (**A**) The longitudinal striations of the bur (200×); (**B**) Most of the dentin tubules were occluded, but several were still patent (1000×).

The scanning electron microscopy (SEM) analysis of the axial dentin surfaces prepared with a high-speed handpiece equipped with a diamond bur is presented in Figure 2. The longitudinal striations of the bur, the areas of open dentinal tubules and the remnants of the smear layer were evident. After pretreatment with the desensitizing paste containing 8% arginine and calcium carbonate and incubation for two weeks, most of the dentin tubules were occluded, although several remained patent (Figure 3A,B).

## 3. Discussion

The essential components of Colgate Sensitive Pro-Relief Desensitizing Paste are arginine, bicarbonate and calcium carbonate. This in-office desensitizing paste has been shown to provide fast and persistent relief from dentin hypersensitivity when it is burnished onto sensitive teeth [14,15,16,17,18]. This effect can be observed after just one professional application due to the creation of 2-µm plugs, which primarily comprise calcium and phosphate [17]. The sensitivity relief lasts for up to 28 days and can reduce the post-preparation and post-cementation sensitivity of vital teeth that serve as abutments for fixed partial dentures, but this treatment may only be effective when the retentive strength is not affected. The current results support the first null hypothesis of the study, which was that pretreatment with Colgate Sensitive Pro-Relief Desensitizing Paste would have no effect on the retentive strength of Lava Y-TZP crown copings cemented to human extracted teeth with RMGIC or SARC. However, the second null hypothesis was rejected because the retentive strength of RMGIC was higher than that of SARC.

Previous studies have shown that the in-office Colgate Sensitive Pro-Relief Desensitizing Paste containing 8% arginine and calcium carbonate had no significant effect on the shear bond strength of composite to enamel [19] or dentin [20,21], implying that dentists can achieve optimal bonding even if a patient has been treated with Colgate Sensitive Pro-Relief to manage dentin hypersensitivity. An extended desensitizing paste application time of 18 or 30 s had no adverse effect on the bonding performance to dentin [20]. The aforementioned studies involved dentin pretreatment with either phosphoric or citric acid to simulate a postoperative sensitivity model. The current study is in agreement with these studies [20,21], although we attempted to mimic the standard clinical procedure without intentionally opening the dentin tubules.

As demonstrated by the SEM micrographs, two weeks after the application of the desensitizing paste, most of the tubules were still occluded (Figure 3B) compared with the post-preparation dentin (Figure 2), and this occlusion had no negative effect on the retentive values obtained with either SARC or RMGIC. SARCs, such as the RelyX U-200 used in the current study, do not contain any bonding agents; therefore, resin sealing or desensitizing agents might compromise the bonding to the dentin. Sailer *et al.* [22] and Stawarczyk *et al.* [23,24] showed that glutaraldehyde/HEMA treatment and resin sealing of the dentin following tooth preparation had a beneficial effect on the shear bond strength of RelyX Unicem, which is the previous generation of the SARC used in the current study. According to the postulated mechanism, the HEMA-pretreated dentin surface allowed the resin cement to wet the dentin surface more effectively, thereby resulting in increased bond strength [22]. The bonding mechanism of RelyX Unicem to the dentin starts with the ionization of methacrylated phosphoric acid during the mixing of the monomers. These negatively charged groups react with calcium and incorporate the smear layer to create an interaction zone without a distinct hybrid layer with resin tags [25]. The plugs created after the application of the Pro-Relief Desensitizing Paste, which primarily comprises calcium and phosphate, can be regarded as part of the smear layer that was not removed after preparation of the dentin by the diamond bur; therefore, they do not interfere with the creation of the interaction zone. The current findings correspond those recently reported by Bavbek *et al.* [7] showing that Colgate Sensitive Pro-Relief did not interfere with the bonding of another SARC (Clearfil SA Cement, Kuraray Co., Osaka, Japan) to dentin. The insignificant increase in the adhesion value was attributed to the glycerin content of the toothpaste [7]. However, it is important to note that we investigated the Pro-Relief in-office paste as opposed to the Pro-Relief toothpaste used by Bavbek *et al.*, which also contains 1450 ppm of sodium monofluorophosphate [7].

The lack of effect that Colgate Sensitive Pro-Relief Desensitizing Paste with 8% arginine and calcium carbonate had on the interaction of RelyX U-200 with the dentin was also verified by the absence of changes in the failure mode, which was mainly adhesive cement-crown. This finding implies that all or most of the dentin was covered by cement. The observation that the retentive strength of RMGIC did not deteriorate after the application of Pro-Relief Desensitizing Paste is in accordance with previous studies demonstrating that the maintenance or removal of the smear layer did not affect the bond strength of RMGIC to the dentin substrate [26]. The retentive ability of RMGIC was previously shown to be unaffected by another desensitizing agent, GC Tooth Mousse [27].

The cements tested in this study were an RMGIC and an SARC. SARCs, which are partially hydrophilic cements, are simple to use and more efficient to handle because they require no pretreatment of the tooth substance [28]. However, in the current study, the retentive values for Y-TZP copings cemented to teeth with or without pretreated dentin surfaces were significantly higher for RMGIC (RelyX Luting 2) than for SARC (RelyX U-200). These findings are in contrast to those of Palacios *et al.* [12], who showed no significant difference in the retentive strengths of Y-TZP ceramic crowns cemented with a composite resin cement with an adhesive agent (Panavia F 2.0), an RMGIC (RelyX Luting) and an SARC (RelyX Unicem). Similarly, Capa *et al.* [29] found no significant difference in the shear bond strength of composite to Y-TZP between SARC (RelyX Unicem) and RMGIC (FujiCem). On the other hand, Sabatini *et al.* [30] reported a higher bond strength with SARC than with RMGIC to various prosthodontic substrates, including Y-TZP. The apparent contradiction between the current and aforementioned studies [12,29,30] might be attributed to the fact that the intaglio surfaces of the Y-TZP crowns in the current study were not sandblasted with 50-µm aluminum oxide particles, which is why the failure mode of U-200 was mainly adhesive cement-crown. An appreciable improvement in the adhesion of RelyX U-200 to zirconia by surface grit blasting was recently reported [31,32].

## 4. Experimental Section

Forty freshly extracted, caries-free, intact molars were collected for this study. All external debris was removed by curettes. The teeth were stored in a germ-free 0.1% thymol solution at room temperature for 1 day and then switched to tap water for another 2 weeks. The roots of the teeth were notched for retention and embedded parallel to the long axis of the tooth by a custom-designed alignment apparatus. Each tooth was suspended in the middle of an aluminum ring and mounted 2 mm apical to the cementoenamel junction (CEJ) in poly(methyl methacrylate) resin (Quick resin, Ivoclar, Schaan, Liechtenstein). At all times, the mounted teeth were stored in tap water at room temperature.

**Figure 4:**
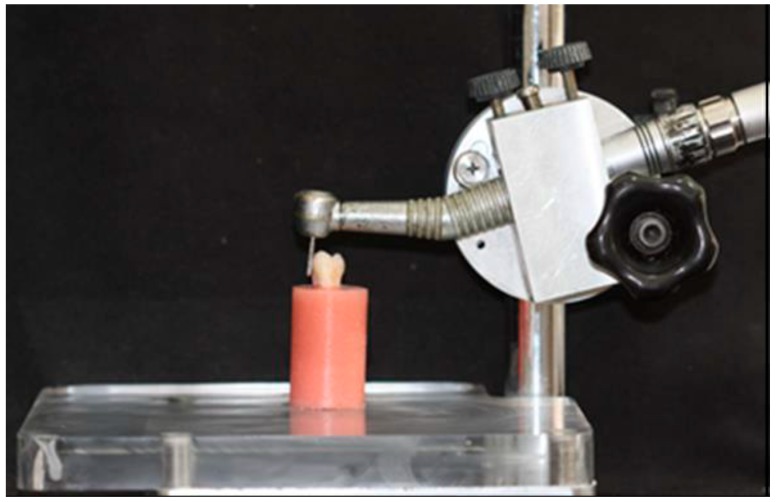
Surveyor-like apparatus for the standardized preparation of mounted extracted teeth.

All teeth were prepared with a standardized protocol to yield an axial height of 5 mm and a 10° taper. The occlusal surface was sectioned perpendicular to the long axis 6 mm above the CEJ with a water-cooled precision saw (Isomet Plus, Buehler, IL, USA). A 0.4-mm, 360° chamfer finish line located 1 mm above the CEJ with a 10° taper was prepared with a rigidly secured, high-speed handpiece equipped with a diamond bur (C_1_-Strauss, Ra’anana, Israel) mounted on a custom-designed, surveyor-like apparatus (Figure 4). A new diamond bur was used for each tooth.

Impressions of the prepared teeth were made using a two-step technique. The preliminary impression was made with a copper ring filled with an acrylic material (Unifast, GC, Alsip, IL, USA). In the second step, a 2-mm acrylic layer was removed from the inner aspect of the ring, and a condensation silicone wash impression material (Xantopren, Heraeus Kulzer, Germany) was injected. To compensate for the difference between room and mouth temperatures, the setting time was doubled. The impressions were poured with type 4 dental stone (Silky Rock, Whipmix, Louisville, KY, USA).

**Figure 5:**
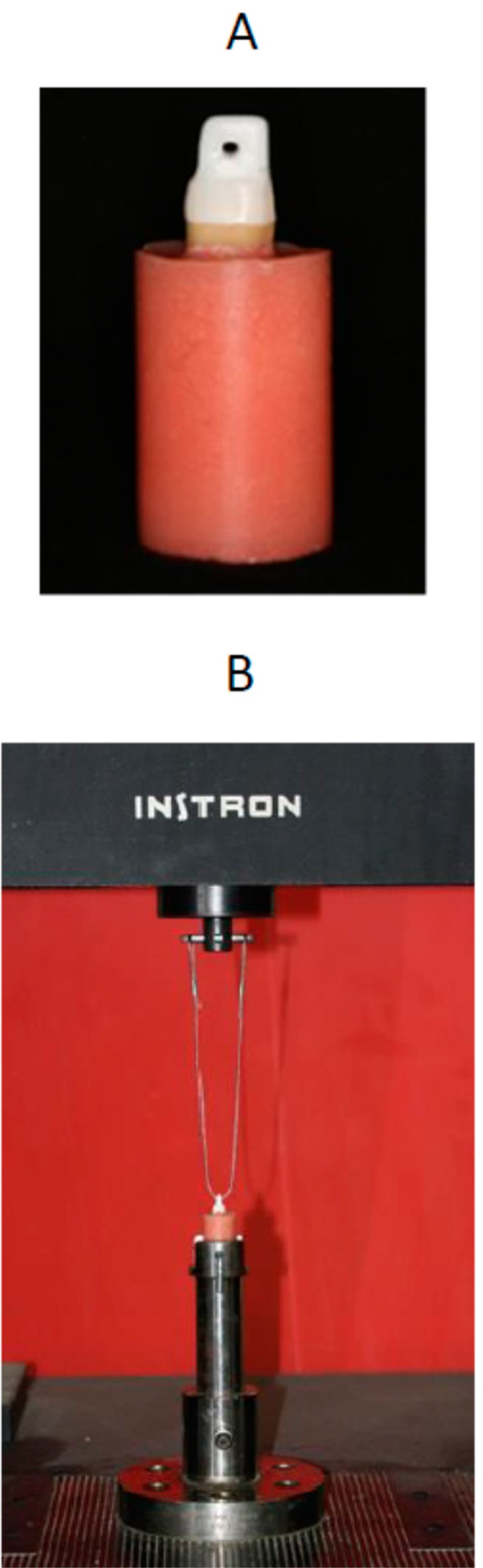
(**A**) Zirconium oxide coping showing the design of the occlusal surface to enable removal after cementation; (**B**) Metal cable connecting the coping with the universal testing machine.

Forty crowns were produced using CAD-CAM technology with Lava frame Y-TZP blocks (3M ESPE, Seefeld, Germany). The Lava CAD-CAM system includes an optical scanner (Lava Scan), a CAM machine (Lava Form) and a sintering oven (Lava Therm). CAD-CAM Y-TZP cores that were 1.0 mm thick with a 50-µm virtual spacer layer were milled at a commercial dental laboratory (Lava milling center, Dental Center, Tel-Aviv). The Lava zirconia copings were designed with a loop (4-mm outer diameter and 2-mm inner diameter) extending coronally from the occlusal surface to facilitate tensile loading (Figure 5A) [33]. The 40 prepared teeth were randomly assigned to two groups (2 × 20). In the first group, the dentin surfaces were pretreated with Colgate Sensitive Pro-Relief Desensitizing Paste using prophy cups with light pressure according to the manufacturer’s recommendations. In the second group, which served as the control group, the dentin surfaces were not pretreated. Y-TZP copings were placed on each tooth, which was then stored at 37 °C under 100% humidity for 2 weeks, resembling a period of reevaluation prior to final cementation. In each group, two luting agents were evaluated (2 × 10): an RMGIC (RelyX Luting 2/3M ESPE) and an SARC (RelyX U-200/3M ESPE). Prior to cementation, the areas of the axial and occlusal surfaces of each prepared tooth were measured as previously described [33]. The intaligo surfaces of the crowns were cleaned with Ivoclean (Ivoclar Vivadent, Schaan, Liechtenstein). The cements were used according to the manufacturer’s recommendations. RelyX U-200 was light-cured for 20 s from each surface by Elipar FreeLight 2 (3M ESPE, St. Paul, MN, USA). Each crown was cemented to its tooth in a standardized manner under a constant load of 10 kg (Force gauge, FG 20, Lutron, Taiwan) for 10 min and allowed to set for 24 h.

To determine whether there were differences between the test groups prior to cementation, two additional examples of prepared teeth from each group were inspected under SEM (Quantum 2000) in high vacuum mode following gold sputter coating. The acquisition conditions were as follows: 25 kV, 90 µA and 200–1000× magnification.

The cemented crown-tooth assemblies were stored in water at 37 °C for 30 days before thermal cycling. After this storage period, the cemented copings were thermally cycled between water temperatures of 5 and 55 °C for 10,000 cycles, which is equivalent to an entire year of clinical physiological aging [34], with a 10-s dwell time (Y. Manes, TA, Israel). After thermal cycling, the crown-tooth assemblies were stored in water at 37 °C for another 30 days and then tested for tensile debonding strength. The crowns were subjected to dislodgment forces through a 1.2-mm diameter metal cable entangled through a coronal loop along the apico-occlusal axis using a universal testing machine (Instron, Model 4502, Instron Corp., Buckinghamshire, UK) at a crosshead speed of 1 mm/min (Figure 5B) until failure. The force at dislodgment was recorded and divided by the total surface area of each preparation to yield the retention value (MPa).

The debonded surfaces of the teeth and crowns were examined microscopically at 10× magnification (M8 Stereomicroscope, Wild Heerbrugg, Switzerland). Each surface of the dentin-crown interface was analyzed separately (five surfaces per tooth). Failure was classified based on the criteria presented in Table 2.

A separate analysis was performed for each matched Y-TZP tooth surface (buccal, lingual, mesial, distal and occlusal). For each category, the number of surfaces was summed and presented as a percentage of all the surfaces for the specific treatment.

### Statistical Analysis

Retentive strength was evaluated using two-way analysis of variance (ANOVA) with repeated measures; cement (*n* = 2) and pretreatment (*n* = 2) were the independent variables. The level of significance was 0.05.

## 5. Conclusions

An 8.0% arginine and calcium carbonate in-office desensitizing paste can be safely used on dentin to reduce post-cementation sensitivity without compromising the retention of Y-TZP crowns cemented with either RMGIC or SARC.

## Figures and Tables

**Table 1:** Means (SD) of the retentive strength (MPa) of the zirconium-oxide crowns for all cementation groups.

Cement Type	Treatment	Sample No.	Mean Retentive Value (MPa)	Standard Deviation
RelyX U-200	−	10	2.29	0.55
	+	10	2.27	0.64
	Total	20	2.28	0.58
RelyX Luting 2	−	10	3.16	0.73
	+	10	2.92	0.84
	Total	20	3.04	0.77
Total	−	20	2.72	0.77
	+	20	2.60	0.80
	Total	40	2.66	0.78

Treatment: − Without pretreatment with Colgate Sensitive Pro-Relief Desensitizing Paste (control); + with pretreatment with Colgate Sensitive Pro-Relief Desensitizing Paste.

**Table 2:** Classification of failure criteria.

Classification	Description	Criteria
1	Cement principally on crown surface	Adhesive cement-dentin
2	Cement principally on dentin surface	Adhesive cement-crown
3	Cement equally distributed on dentin and crown surfaces	Cohesive cement
4	Mixed mode	Adhesive and cohesive cement
5	Fracture of the tooth	Cohesive dentin
